# Folate deficiency correlates with severity of primary biliary cholangitis via modulating key regulatory genes

**DOI:** 10.3389/fnut.2026.1838352

**Published:** 2026-06-03

**Authors:** Yujie Yan, Qie Mu, Bonan Li, Yuqing Sun, Hong Yang, Zhiqin Huang, Baofeng Yu, Li Zhang, Huifang Huang

**Affiliations:** 1 Department of Gastroenterology, The First Hospital of Shanxi Medical University, Taiyuan, Shanxi, China; 2 Department of Biochemistry and Molecular Biology, Shanxi Medical University, Taiyuan, Shanxi, China; 3 School of Public Health, Shanxi Medical University, Taiyuan, Shanxi, China; 4 Department of Hepatobiliary Surgery and Liver Transplant Center, The First Hospital of Shanxi Medical University, Taiyuan, Shanxi, China; 5 Third Hospital of Shanxi Medical University, Shanxi Bethune Hospital, Taiyuan, China; 6 Department of Nutrition, The First Hospital of Shanxi Medical University, Taiyuan, Shanxi, China

**Keywords:** biomarker, disease progression, folate, primary biliary cholangitis, transcriptomic

## Abstract

**Objective:**

To investigate the association between serum folate and Primary biliary cholangitis (PBC) progression through clinical, transcriptomic, and protein-level analyses.

**Methods:**

Serum folate levels were measured in 90 patients with PBC and 90 healthy controls. RNA-sequencing (RNA-seq) was performed on three subgroups (PBC patients with normal folate, PBC patients with low folate, and controls), and six candidate genes were validated by enzyme-linked immunosorbent assay (ELISA).

**Results:**

Serum folate was significantly decreased in PBC patients, with further reduction in late-stage disease (*p* < 0.05), and negatively correlated with ALT, AST, GGT, IgG, and LSM. Combined detection of folate with DBIL and LSM achieved excellent diagnostic performance for advanced PBC (AUC = 0.974). Transcriptome analysis identified 2,363–5,648 DEGs enriched in metabolism and immune pathways. ELISA confirmed dysregulation of AHRR, MXD1, ARID3B, DECR2, and P2RY14 in PBC groups, with expression patterns linked to folate status.

**Conclusion:**

Serum folate is inversely correlated with PBC severity and may serve as a potential predictive biomarker. Folate deficiency likely contributes to PBC pathogenesis by modulating key genes, including ARID3B and MXD1.

## Introduction

Primary biliary cholangitis (PBC) is a chronic, progressive autoimmune liver disease characterized by immune-mediated destruction of the intrahepatic small bile ducts. This process leads to cholestasis and hepatic fibrosis, ultimately progressing to cirrhosis and liver failure (1, 2). Although the exact etiology and pathogenesis of PBC remain incompletely understood, they are currently believed to be closely associated with genetic susceptibility, environmental triggers, and immune dysregulation (3). PBC demonstrates a marked female predominance, with an incidence in middle-aged women approximately four to six times higher than in men. Globally, the estimated annual incidence and prevalence of PBC are 1.76 per 100,000 person-years and 14.60 per 100,000, respectively. Both have exhibited a steady upward trend over the past three decades, making PBC a leading cause of end-stage liver disease (4–6). Ursodeoxycholic acid (UDCA) is the first-line pharmacotherapy and has been shown to improve bile acid profiles and slow disease progression in a subset of patients. However, approximately 40% of patients exhibit an inadequate response to UDCA and remain at risk for disease progression (4, 7). Therefore, identifying novel modifiable factors that influence the onset and progression of PBC is of great clinical importance for improving patient outcomes and prognosis.

Folic acid (FA) is a water-soluble B vitamin. First purified and named by Lucy Wills in 1931, it is one of the essential vitamins for human growth and reproduction (8). Folate serves as a carbon unit donor involved not only in DNA synthesis and repair but also in epigenetic regulation, immune cell differentiation, and the maintenance of oxidative stress balance (9, 10). In recent years, the relationship between abnormal folate metabolism and autoimmune diseases has emerged as a key area of research. For example, in patients with rheumatoid arthritis (RA), reduced serum folate levels have been shown to correlate with disease activity. Folate deficiency may impair DNA methylation and immune cell function, potentially exacerbating inflammatory responses and metabolic disturbances, while also increasing the risk of mortality in this population (11, 12). Similarly, in systemic lupus erythematosus (SLE), folate deficiency induces abnormal DNA methylation, leading to the overexpression of inflammatory lymphocytes and promoting disease progression (13, 14). The liver is the main storage site for folate, and emerging evidence links low serum folate levels to hepatic dysfunction and the progression of chronic liver diseases such as metabolic fatty liver disease, alcoholic liver disease, and fibrosis (15, 16).

However, the role of folate in PBC remains systematically unexplored. Specifically, it is unknown whether serum folate levels correlate with disease severity in PBC and whether folate deficiency contributes to PBC progression by modulating specific molecular pathways. Folate is a key cofactor in one-carbon metabolism, participating in methylation reactions, nucleotide synthesis, redox homeostasis, and immune cell regulation. Folate deficiency impairs one-carbon metabolic flux, leading to insufficient DNA methylation and hyperhomocysteinemia. This potentially forms a molecular network driving PBC progression through multiple mechanisms, including (1) epigenetic dysregulation resulting in aberrant expression of key genes such as ARID3B and MXD1. (2) activation of NF-κB mediated inflammatory signaling and the TGF-*β*/Smad fibrotic pathway. (3) inhibition of the AhR and NRF2 antioxidant pathways thereby exacerbating oxidative damage to cholangiocytes. (4) modulation of purinergic receptor P2RY14 expression and immune cell chemotaxis. Notably, hepatic dysfunction and enterohepatic circulation disturbances inherent to PBC can conversely reduce folate levels, establishing a bidirectional vicious cycle in which folate deficiency leads to PBC progression, which in turn reduces folate absorption and further aggravates folate deficiency. Although dysregulation of folate metabolism has been implicated in the development of alcoholic liver disease and non-alcoholic fatty liver disease, its role in PBC remains systematically unexplored. Specifically, it remains unknown whether serum folate levels correlate with disease severity in PBC and whether folate deficiency contributes to PBC progression through specific molecular pathways. Therefore, we hypothesized that serum folate levels may serve as a potential biomarker of PBC severity and that folate deficiency could contribute to disease pathogenesis by dysregulating specific genes.

This study aimed to investigate the relationship between serum folate levels and disease progression in patients with PBC. Transcriptomic analysis was performed to identify differentially expressed genes and enriched pathways in PBC patients stratified by folate status, with the goal of uncovering underlying molecular mechanisms. These key findings were further validated at the protein level, elucidating the role of folate in the pathogenesis and progression of PBC and providing potential novel insights for therapeutic intervention.

## Materials and methods

### Study subjects

This retrospective study enrolled 90 patients diagnosed with PBC at the First Hospital of Shanxi Medical University between January 2024 and December 2025. The diagnosis was established according to the Guidelines for the Diagnosis and Treatment of Primary Biliary Cholangitis (17). These patients were assigned to the PBC group. A control group consisted of 90 healthy individuals who underwent routine physical examinations at the same hospital during the identical period, which required meeting any two of the following three criteria. The inclusion criteria were as follows: (1) biochemical evidence of cholestasis, primarily characterized by elevated alkaline phosphatase (ALP) and gamma-glutamyl transferase (GGT), with imaging studies ruling out extrahepatic or large intrahepatic bile duct obstruction. (2) positivity for anti-mitochondrial antibodies (AMA) or AMA-M2 subtype, or presence of other PBC-specific autoantibodies (e.g., anti-gp210 and anti-sp100). (3) histological confirmation of non-suppurative destructive cholangitis and destruction of the small bile ducts.

Patients were excluded if they met any of the following criteria: (1) presence of other liver diseases (e.g., viral hepatitis, alcoholic liver disease). (2) complicated by severe cardiovascular, cerebrovascular, or renal diseases. (3) pregnancy or lactation. (4) history of folic acid supplementation within three months prior to enrollment. (5) receipt of other treatments that could potentially affect folate levels. (6) unclear diagnosis or incomplete clinical data. According to serum folate concentrations, PBC patients were further divided into a normal folate group (6.8–36.3 nmol/L) and a low folate group (< 6.8 nmol/L). This study was conducted in accordance with the Declaration of Helsinki. The study protocol was approved by the Medical Ethics Committee of the First Hospital of Shanxi Medical University, and written informed consent was obtained from all participants.

### Sample size estimation

The primary outcome was the difference in serum folate levels between PBC patients and healthy controls. Based on our pilot data, the estimated mean difference was 1.2 ng/mL with a common standard deviation of 2.4 ng/mL, corresponding to a medium effect size (Cohen’s d = 0.5). Using a two-sample t-test formula with a two-sided *α* of 0.05 and a power (1-*β*) of 0.80, the minimum required sample size was 64 participants per group (calculated using G*Power 3.1). Our study enrolled 90 PBC patients and 90 healthy controls, exceeding the minimum requirement and thus providing adequate statistical power.

### Clinical data collection and index detection

The following data were collected from all subjects: (1) General characteristics: gender, age, and other basic demographic information. (2) Laboratory parameters: red blood cell count (RBC), hemoglobin (Hb), platelet count (PLT), aspartate aminotransferase (AST), alanine aminotransferase (ALT), gamma-glutamyl transferase (GGT), alkaline phosphatase (ALP), total bilirubin (TBIL), direct bilirubin (DBIL), indirect bilirubin (IBIL), albumin (ALB), uric acid (UA), blood urea nitrogen (BUN), serum creatinine (SCr), total cholesterol (TC), triglycerides (TG), high-density lipoprotein cholesterol (HDL-C), low-density lipoprotein cholesterol (LDL-C), immunoglobulin G (IgG), immunoglobulin A (IgA), immunoglobulin M (IgM), AMA-M2 antibody, anti-gp210 antibody, anti-sp100 antibody, and serum folate level. (3) Imaging and functional examination results: abdominal color Doppler ultrasound and liver transient elastography (FibroScan).

The natural course of PBC was classified into the following four stages, as previously described (18): Stage I, characterized by positive serum AMA without significant biochemical abnormalities. Stage II, defined by abnormal biochemical parameters in the absence of overt clinical symptoms. Stage III, marked by the presence of clinical symptoms such as fatigue and pruritus, and Stage IV, distinguished by the development of severe complications, including gastrointestinal bleeding, ascites, or hepatic encephalopathy. Based on this staging system, patients in the PBC group were further stratified into two subgroups: the early-stage PBC group (Stages I–II, *n* = 22) and the late-stage PBC group (Stages III–IV, *n* = 68).

### RNA isolation, library construction, and sequencing

From each of the three groups (normal-folate PBC, low-folate PBC, and control), seven patients were randomly selected. Approximately 5 mL of peripheral venous blood was collected into EDTA anticoagulant tubes, mixed with TRIzol, immediately frozen in liquid nitrogen for 0.5 h, and stored at −80 °C until analysis.

Total RNA was extracted using TRIzol reagent (Thermo Fisher, 15,596,018) following the manufacturer’s protocol. RNA quantity and purity were assessed using a NanoDrop ND-1000 (NanoDrop, Wilmington, DE, United States) and a BioAnalyzer 2,100 (Agilent, CA, USA). Only samples meeting the following criteria were used for library construction: RNA concentration>50 ng/μL, RIN > 7.0, and total RNA > 1 μg. Total RNA was extracted using Trizol reagent (ThermoFisher,15596018) following the manufacturer’s procedure. The total RNA quantity and purity were analysis of NanoDrop ND-1000 (NanoDrop, Wilmington, DE, United States) and Bioanalyzer 2,100 (Agilent, CA, United States), high-quality RNA samples with >50 ng/μL, RIN number>7.0 and total RNA > 1 μg were used to construct sequencing library. The poly A-tail mRNA was enriched twice using Dynabeads Oligo (dT) (Thermo Fisher, cat.25–61005). Following purification, the mRNA was fragmented into short fragments using NEBNextR Magnesium RNA Fragmentation Module at 94 °C for 5–7 min (cat. E6150S, United States). Then the cleaved RNA fragments were reverse-transcribed to synthesize first-Strand cDNA by Invitrogen SuperScriptTM II Reverse Transcriptase (cat.1896649, CA, United States), which were next used to synthesise second-stranded DNAs with *E. coli* DNA polymerase I (NEB, cat.m0209, United States), RNase H (NEB, cat. m0297, United States) and dUTP Solution (Thermo Fisher, cat. R0133, CA, United States). An A-base was then added to the blunt ends of each strand, preparing them for ligation to the indexed adapters. Each adapter contained a T-base overhang for ligating the adapter to the A-tailed fragmented DNA. The Dynabeads Oligo are used to screen and purify the fragment size. The double strands were digested with UDG enzyme (NEB, cat. m0280, MA, United States), and the ligated products were amplified with PCR by the following conditions: pre-denaturation at 95 °C for 3 min, denaturation at 98 °C for a total of 8 cycles, each cycle lasting for 15 s, annealing at 60 °C for 15 s, extension at 72 °C for 30 s, and finally extension at 72 °C for 5 min to form a library with a fragment size of 300 bp ± 50 bp (strand-specific library). Finally, we used the Illumina NovaseqTM 6,000 (LC Bio Technology CO., Ltd., Hangzhou, China) for paired-end sequencing according to standard operating procedures, with a sequencing mode of PE150.

Raw reads were filtered to remove adapter sequences and low-quality reads (Q < 20) using fastp (version 0.23.2). Clean reads were aligned to the human reference genome (GRCh38) using HISAT2 (version 2.2.1). Gene-level read counts were quantified using feature Counts (version 2.0.3). Differential expression analysis was performed using DESeq2 (version 1.38.3) with a false discovery rate (FDR) < 0.05 and |log₂ fold change| ≥ 1 as the threshold for significant DEGs. Functional enrichment analyses, including Gene Ontology (GO) and Kyoto Encyclopedia of Genes and Genomes (KEGG) pathway analyses, were conducted using clusterProfiler (version 4.6.2) with a significance cutoff of FDR < 0.05.

### Enzyme-linked immunosorbent assay (ELISA) analysis

The ELISA kit provided by Vankewei Company was utilized to detect the protein expression levels of ARID3B, MXD1, AHRR, P2RY14, DECR2, and SPIB in human serum samples. All experimental procedures strictly followed the standard protocol provided by the kit manufacturer. To ensure the reliability and reproducibility of the results, all samples were analyzed in duplicate.

### Statistical analysis

All data were derived from at least three independent biological replicates. Statistical analyses were performed using SPSS 27.0. Normally distributed continuous variables were expressed as mean ± standard deviation (x̅±s) and compared between groups using independent samples *t*-tests. Non-normally distributed data were presented as median (interquartile range) and analyzed using nonparametric tests. Categorical variables were expressed as frequencies or percentages and compared using the chi-square test. Correlations were assessed by Spearman’s rank correlation analysis. Binary logistic regression was employed to identify independent risk factors for PBC, and receiver operating characteristic (ROC) curves were constructed to evaluate diagnostic performance. A two-tailed *p* < 0.05 was considered statistically significant.

## Results

### General characteristics of the study subjects

As shown in Table 1, the PBC group in this study included 90 patients, comprising 20 males and 70 females, with a mean age of 59.66 ± 11.72 years. The control group included 90 patients, comprising 28 males and 62 females, with a mean age of 57.00 ± 12.68 years. There were no statistically significant differences between the two groups in terms of age or gender (*p* > 0.05). Compared with the control group, the PBC group showed significantly lower levels of RBC, Hb, ALB, SCr, UA, TC, TG, HDL-C, LDL-C, and folate, while ALT, AST, GGT, ALP, TBIL, DBIL, and IBIL were significantly elevated. All differences were statistically significant (*p* < 0.05). No statistically significant differences were observed in the remaining biochemical parameters.

**Table 1:** Comparison of general clinical data between the PBC group and the control group.

Characteristics	Control	PBC	*P*
Age (years)	57.00 ± 12.68	59.66 ± 11.72	0.146
Female gender (%)	62 (68.9%)	70 (77.8%)	0.178
RBC (10^9/L)	4.78 ± 0.48	3.67 ± 0.86	0.000
Hb (g/L)	147.00 (134.00, 157.00)	109.00 (86.75, 130.25)	0.000
PLT (10^9/L)	235.00 (197.75, 270.00)	145.50 (77.00, 204.00)	0.000
ALT (U/L)	17.00 (13.00, 24.00)	26.00 (15.75, 51.00)	0.000
AST (U/L)	21.00 (18.00, 25.00)	38.50 (25.00, 67.25)	0.000
GGT (U/L)	20.50 (17.00, 33.00)	62.00 (28.00, 185.50)	0.000
ALP (U/L)	79.00 (66.00, 91.50)	131.00 (91.50, 217.25)	0.000
TBIL (μmol/L)	13.35 (10.68, 16.95)	22.70 (14.85, 31.95)	0.000
DBIL (μmol/L)	2.30 (1.70, 2.85)	7.10 (3.90, 12.50)	0.000
IBIL (μmol/L)	11.15 (9.08, 14.00)	14.50 (10.73, 20.68)	0.000
ALB (g/L)	45.96 ± 2.87	34.65 ± 7.43	0.000
BUN (mmol/L)	4.80 (4.07, 5.59)	4.53 (3.74, 5.92)	0.653
SCr (μmol/L)	62.45 (52.43, 73.55)	53.65 (46.75, 62.15)	0.000
UA (μmol/L)	295.00 (250.75, 388.00)	282.50 (220.25, 350.00)	0.028
TC (mmol/L)	4.71 (4.16, 5.31)	3.70 (2.86, 4.70)	0.000
TG (mmol/L)	1.44 (0.99, 2.06)	1.02 (0.78, 1.37)	0.000
HDL-C (mmol/L)	1.34 (1.13, 1.55)	1.15 (0.91, 1.40)	0.000
LDL-C (mmol/L)	2.95 (2.42, 3.37)	2.27 (1.53, 2.98)	0.000
FA (nmol/L)	21.38 (18.86, 24.07)	17.25 (11.93, 25.93)	0.000

As shown in Table 2, compared with the early-stage PBC group, patients in the advanced-stage group exhibited significantly higher age and a lower proportion of females, with both differences being statistically significant (*p* < 0.05). However, no statistically significant difference in BMI was observed between the two groups (*p* > 0.05). Additionally, compared with the early-stage PBC group, the late-stage group exhibited significantly lower levels of RBC, Hb, PLT, ALB, TC, HDL-C, LDL-C, CAP, and folate, while showing significantly higher levels of AST, ALP, TBIL, DBIL, IBIL, IgG, IgA, and LSM. All differences were statistically significant (*p* < 0.05). No statistically significant differences were observed in the remaining biochemical parameters.

**Table 2:** Comparison of clinical data between early-stage and late-stage PBC groups.

Characteristics	Early stage	Advanced stage	*p*
Age (years)	51.23 ± 12.86	62.38 ± 9.99	0.000
Female gender (%)	21 (95.5%)	49 (72.1%)	0.046
BMI (kg/m^2^)	23.99 ± 3.06	22.59 ± 3.79	0.086
RBC (10^9/L)	4.22 ± 0.75	3.49 ± 0.82	0.000
Hb (g/L)	126.27 ± 23.98	102.18 ± 28.35	0.001
PLT (10^9/L)	214.50 (188.75, 243.50)	105.50 (68.50, 170.50)	0.000
ALT (U/L)	24.00 (13.00, 34.75)	27.50 (16.50, 51.75)	0.217
AST (U/L)	24.50 (19.75, 30.75)	45.50 (30.00, 84.75)	0.000
GGT (U/L)	52.00 (27.50, 104.25)	67.00 (28.00, 210.00)	0.357
ALP (U/L)	94.50 (75.50, 140.00)	137.50 (95.50, 249.75)	0.005
TBIL (μmol/L)	12.95 (9.90, 19.65)	24.45 (17.30, 38.00)	0.000
DBIL (μmol/L)	2.65 (1.95, 5.58)	8.40 (5.93, 17.40)	0.000
IBIL (μmol/L)	9.80 (6.60, 13.83)	16.05 (12.28, 23.38)	0.000
ALB (g/L)	42.70 ± 5.24	32.04 ± 6.04	0.000
BUN (mmol/L)	4.31 (3.60, 5.47)	4.62 (3.74, 6.37)	0.338
SCr (μmol/L)	52.30 (46.75, 58.88)	54.40 (46.25, 65.75)	0.350
UA (μmol/L)	255.50 (198.63, 344.75)	284.00 (221.75, 362.00)	0.414
TC (mmol/L)	4.57 (4.06, 5.03)	3.49 (2.72, 4.49)	0.002
TG (mmol/L)	1.24 (0.80, 1.76)	0.96 (0.77, 1.34)	0.217
HDL-C (mmol/L)	1.35 (1.25, 1.54)	1.06 (0.85, 1.34)	0.002
LDL-C (mmol/L)	2.43 (2.25, 3.15)	2.09 (1.45, 2.79)	0.013
IgG (g/L)	15.47 ± 3.99	18.30 ± 6.23	0.016
IgM (g/L)	2.78 (1.68, 3.63)	2.59 (1.47, 3.85)	0.855
IgA (g/L)	2.64 (2.19, 3.13)	3.68 (2.57, 4.78)	0.001
AMA-M2 positive	17 (77.3%)	57 (83.8%)	0.706
gp210 positive	8 (36.4%)	32 (47.1%)	0.380
sp100 positive	4 (18.2%)	7 (10.3%)	0.544
CAP (db/m)	244.82 ± 52.30	214.96 ± 38.47	0.020
LSM (kPa)	7.10 (5.95, 8.95)	28.65 (18.25, 42.03)	0.000
FA (nmol/L)	21.38 (15.80, 26.65)	16.45 (10.63, 24.74)	0.032

### Correlation between serum folate levels and primary biliary cholangitis and its diagnostic value in advanced disease

**Figure 1:**
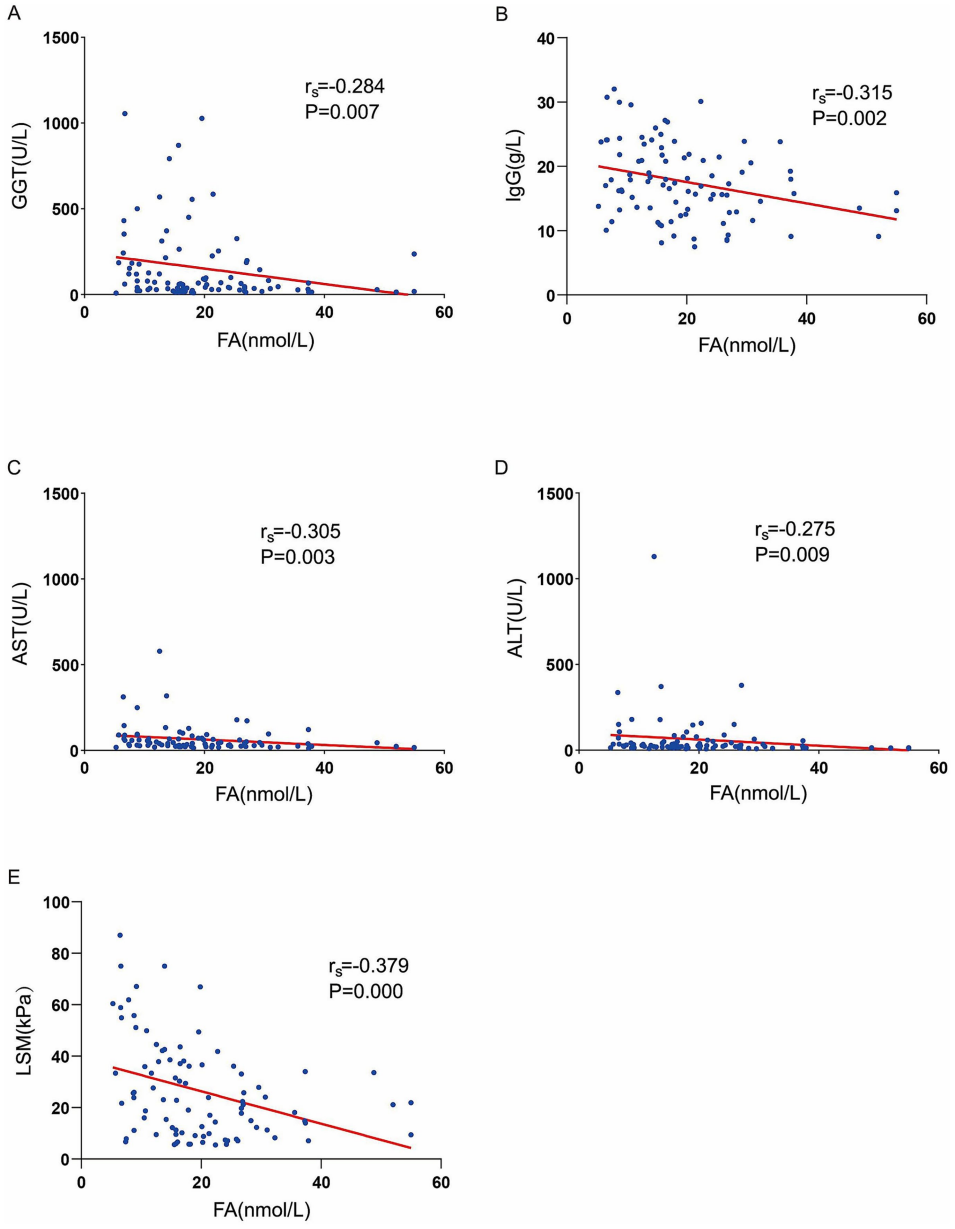
Correlation analysis between FA and laboratory indicators. **(A)** FA concentrations were negatively correlated with GGT (*r* = −0.284, *p* = 0.007). **(B)** FA concentrations were negatively correlated with IgG (*r* = −0.315, *p* = 0.002). **(C)** FA concentrations were negatively correlated with AST (*r* = −0.305, *p* = 0.003). **(D)** FA concentrations were negatively correlated with ALT (*r* = −0.275, *p* = 0.009). **(E)** FA concentrations were negatively correlated with LSM (*r* = −0.379, *p* < 0.001).

The correlation between serum folate levels and laboratory indicators in PBC patients was evaluated. Serum folate levels in PBC patients showed negative correlations with ALT, AST, GGT, IgG, and LSM (correlation coefficients r = −0.275, −0.305, −0.284, −0.315, and −0.379, respectively, *p* < 0.05, Figure 1). No significant correlations were observed with other laboratory parameters (Table 3).

**Table 3:** Correlation analysis between FA and laboratory indicators.

Characteristics	*r*	*P*
ALT	−0.275	0.009
AST	−0.305	0.003
GGT	−0.284	0.007
ALP	−0.198	0.061
ALB	0.173	0.103
TBIL	−0.191	0.072
DBIL	−0.179	0.091
IBIL	−0.175	0.099
IgG	−0.315	0.002
IgM	0.001	0.994
IgA	−0.138	0.194
AMA-M2 positive	−0.045	0.672
gp210 positive	−0.096	0.366
sp100 positive	−0.018	0.8664
CAP	0.087	0.413
LSM	−0.379	0.000

To identify independent factors associated with advanced PBC, binary logistic regression analysis was performed with advanced disease status as the dependent variable. The independent variables included ALP, DBIL, folate, and LSM. All of these variables showed significant differences in the preceding univariate analysis. As presented in Table 3, folate emerged as a protective factor against advanced PBC, while DBIL and LSM were identified as significant risk factors.

To evaluate the predictive value of individual markers for advanced PBC, ROC curve analysis was performed. As shown in Table 4, DBIL, folate, and LSM each demonstrated predictive value for advanced PBC, with optimal cutoff values of 4.40 μmol/L, 15.37 nmol/L, and 9.75 kPa, respectively. The corresponding areas under the curve (AUC) were 0.885, 0.652, and 0.971, with sensitivities of 86.8, 45.6, and 97.1%, and specificities of 72.7, 90.9, and 86.4%, respectively. Notably, the combined detection of folate, DBIL, and LSM yielded significantly improved diagnostic efficacy for advanced PBC compared to any single marker alone, achieving a sensitivity of 91.2%, a specificity of 95.5%, and an AUC of 0.974 (Table 5; Supplementary Figure 1).

**Table 4:** Binary logistic regression analysis of ALP, DBIL, FA, and LSM with the risk of developing advanced PBC.

Factors	B	S.E	OR (95%CI)	*P*
ALP	0.010	0.007	1.010(0.996–1.024)	0.174
DBIL	0.565	0.255	1.760(1.068–2.902)	0.027
FA	−0.200	0.092	0.818(0.683–0.981)	0.030
LSM	0.680	0.246	1.973(1.219–3.194)	0.006

The Hosmer–Lemeshow test showed χ^2^ = 1.590, *p* = 0.979 > 0.05, indicating a good goodness of fit for the model.

**Table 5:** Predictive value of DBIL, FA, and LSM for advanced PBC.

Factors	AUC	*P*	95%CI	Cutoff value	Sensitivity (%)	Specificity (%)
DBIL	0.885	0.000	0.803, 0.967	4.40	86.8	72.7
FA	0.652	0.032	0.527, 0.778	15.37	45.6	90.9
LSM	0.971	0.000	0.935, 1.000	9.75	97.1	86.4
Combined detection	0.974	0.000	0.947, 1.000	0.66	91.2	95.5

Combined detection refers to the combined detection of DBIL, FA, LSM.

### Differential gene expression analysis

**Figure 2:**
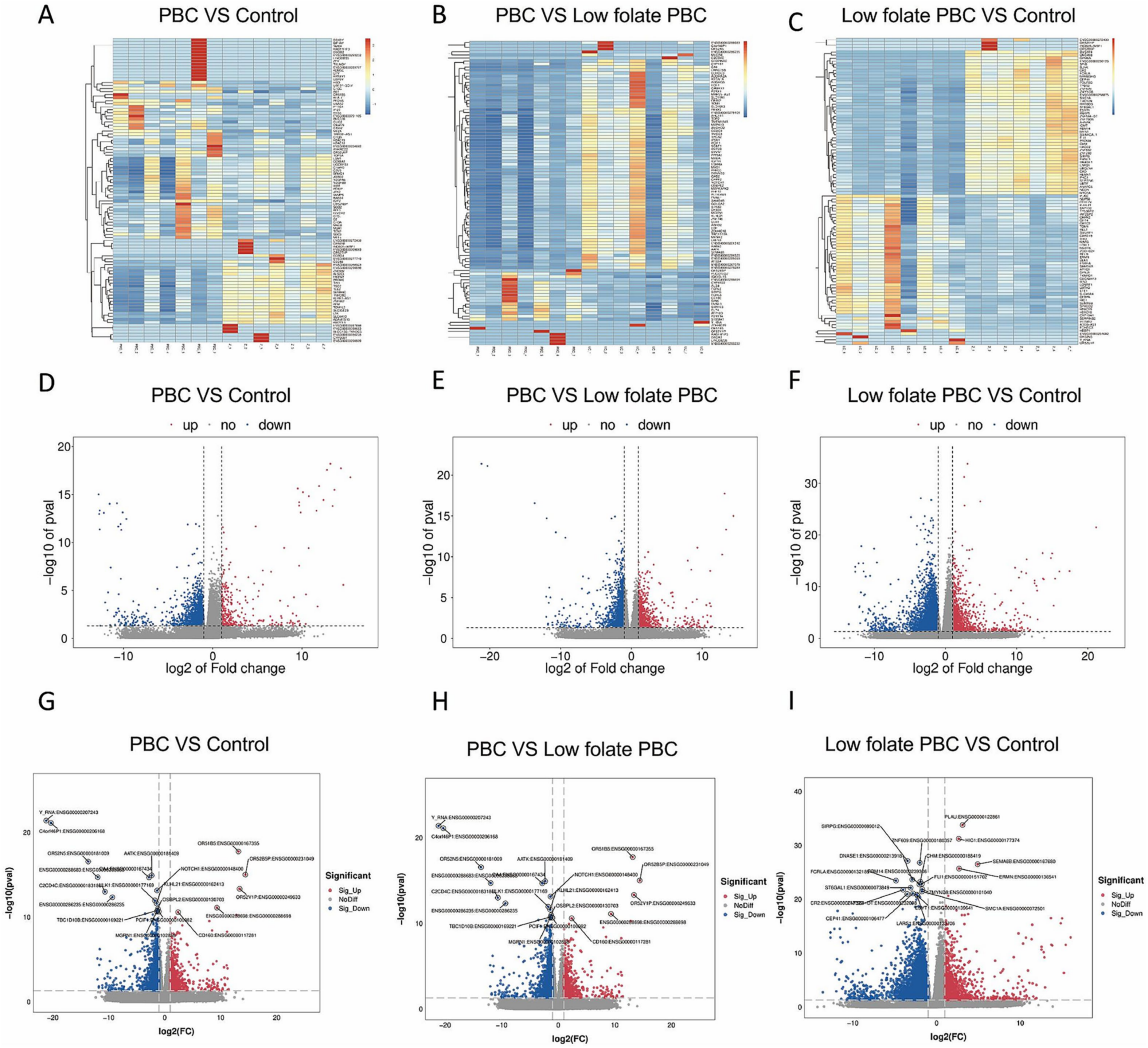
Differential gene expression analysis. **(A)** Heatmap depicting the expression levels of DEGs between the PBC group and the control group. Red and blue represent high and low expression, respectively. **(B)** Heatmap depicting the expression levels of DEGs between the PBC group and the low folate PBC group. **(C)** Heatmap depicting the expression levels of DEGs between the low folate PBC group and the control group. **(D)** Volcano plot showing the distribution of DEGs between the PBC group and the control group. The X-axis represents the log_2_fold change (log_2_FC), and the Y-axis represents the -log_10_*p* value. Red, blue, and gray dots denote significantly upregulated, downregulated, and non-significant genes, respectively. **(E)** Volcano plot showing the distribution of DEGs between the PBC group and the low folate PBC group. **(F)** Volcano plot showing the distribution of DEGs between the low folate PBC group and the control group. **(G–I)** Volcano plots highlighting the top 20 most significantly differentially expressed genes (ranked by *p* value) for the comparisons shown in **(D–F)**, respectively.

Hierarchical clustering analysis revealed distinct gene expression profiles among the three groups (Figures 2A–C). In the normal-folate PBC vs. Control comparison, DEGs exhibited clear clustering patterns with good reproducibility, while comparisons involving Low-folate PBC groups showed substantial differences, suggesting folate deficiency may exacerbate transcriptomic alterations in PBC patients. Volcano plots illustrated the distribution of DEGs across three pairwise comparisons (Figures 2D–F). The normal-folate PBC versus control comparison identified 2,363 DEGs (301 up, 2,062 down), the normal-folate PBC versus low-folate PBC comparison identified 2,343 DEGs (791 up, 1,552 down), and the low-folate PBC versus control comparison identified 5,648 DEGs (1,070 up, 4,578 down). The top 20 most significant DEGs were selected from each comparison (Figures 2G–I), with key genes including OR51B5, RPS4Y1, NOTCH1, CD160, SIRPG, PLAU, and others involved in immune regulation, transcriptional control, and protein degradation. Based on these analyses, six key candidates—ARID3B, MXD1, AHRR, P2RY14, DECR2, SPIB—were selected for serum protein validation by ELISA to explore their roles in PBC pathogenesis and folate-mediated regulation.

### ELISA validation of candidate genes

**Figure 3:**
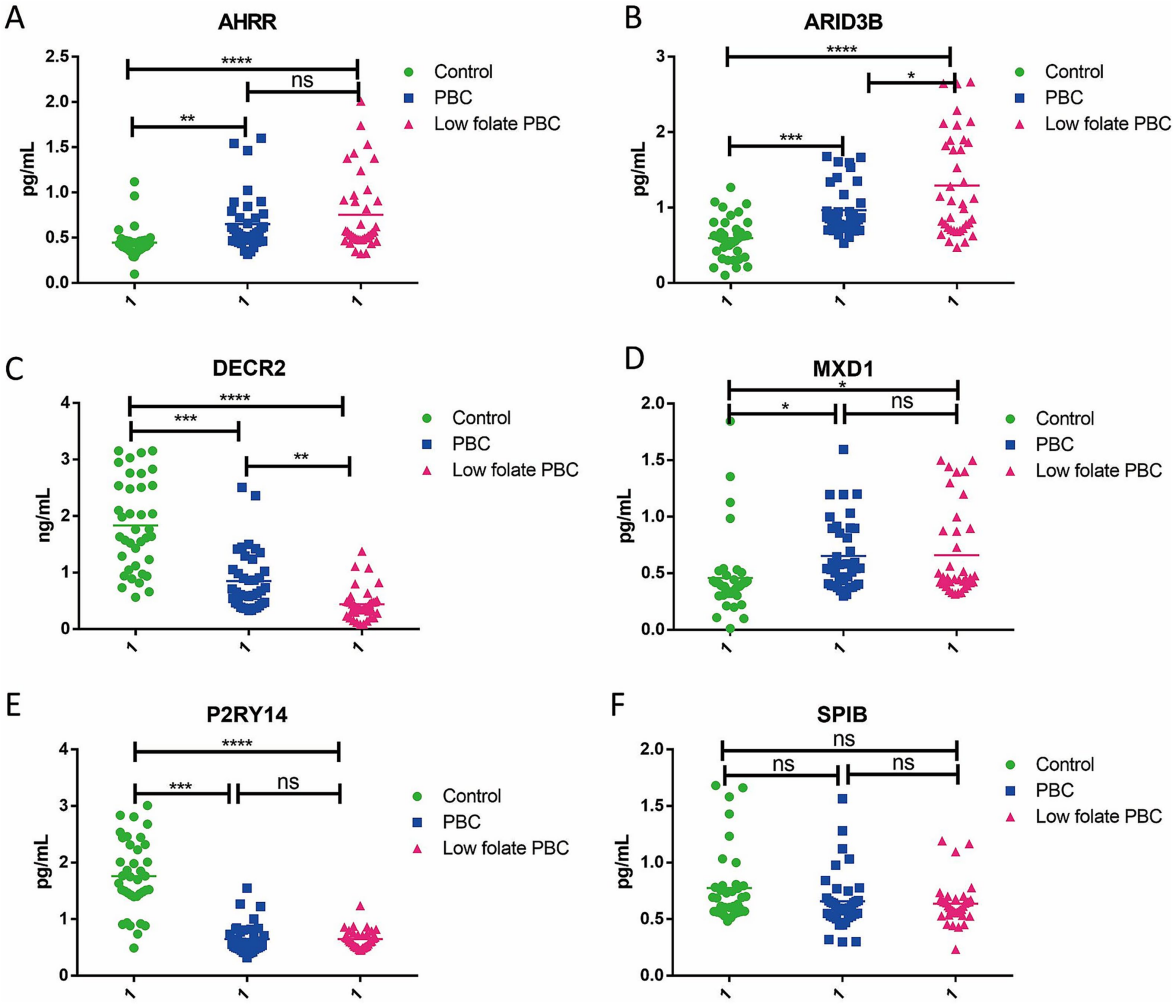
Serum protein levels of AHRR, ARID3B, DECR2, MXD1, P2RY14, and SPIB in the control group, PBC group, and low folate PBC group **(A–F)**. **(A)** AHRR (*n* = 38). **(B)** ARID3B (*n* = 38). **(C)** DECR2 (*n* = 38). **(D)** MXD1 (*n* = 38). **(E)** P2RY14 (*n* = 38). **(F)** SPIB (*n* = 38). *p < 0.05; **p < 0.01; ***p < 0.001; ****p < 0.0001.

ELISA analysis revealed distinct serum protein expression patterns among the three groups. AHRR and MXD1 levels were significantly elevated in both PBC subgroups versus controls, with no difference between folate-stratified PBC groups (Figure 3). ARID3B showed progressive elevation across groups, with significantly higher levels in low-folate PBC versus normal-folate PBC and controls, and in normal-folate PBC versus controls. Conversely, DECR2 and P2RY14 levels were highest in controls, with significant reductions in both PBC subgroups. DECR2 was additionally lower in low-folate versus normal-folate PBC. SPIB expression did not differ significantly among groups.

## Discussion

PBC is a chronic autoimmune liver disease that can progress to cirrhosis and liver failure (19). While the incidence of PBC has remained relatively stable, its prevalence is rising globally, underscoring the need for novel modifiable factors influencing its progression. The liver is the primary storage site for folate, a key player in one-carbon metabolism, DNA synthesis, and epigenetic regulation (20). Folate deficiency has been implicated in the pathogenesis of various liver diseases, yet its specific role in PBC remains poorly understood. In this study, we systematically investigated the association between serum folate levels and PBC progression through an integrated approach combining clinical data, transcriptomic profiling, and protein-level validation. We revealed that low serum folate is significantly associated with advanced PBC, may influence disease pathogenesis by modulating key genes and pathways, and holds potential clinical value as a biomarker.

Several novel findings emerge from this study. First, this is the first investigation to integrate clinical phenotyping, transcriptomic profiling, and protein validation to explore the relationship between folate deficiency and PBC, moving beyond simple correlations with routine biochemical markers. Second, we provide the first molecular link between folate status and PBC pathogenesis: RNA-seq identified differentially expressed genes enriched in immune and metabolic pathways, and ELISA validated key candidates (ARID3B, MXD1, AHRR, P2RY14, DECR2). Notably, the paralog of ARID3B is a known PBC susceptibility gene, and MXD1, a negative regulator of the MYC network, may affect cholangiocyte proliferation-apoptosis balance. Third, we established a composite diagnostic model (folate + DBIL + LSM) for advanced PBC, achieving an AUC of 0.974, which significantly outperforms single indicators and offers a practical early risk stratification tool. Fourth, we propose a bidirectional vicious cycle wherein folate deficiency drives PBC progression, which in turn reduces folate absorption and aggravates deficiency, providing a theoretical basis for folate supplementation as an adjunctive therapy. In summary, beyond validating the association between low folate and PBC severity, our study provides novel multi-omics insights into underlying mechanisms and a promising predictive model with translational potential.

Consistent with previous reports (21), the retrospective analysis demonstrated significantly lower serum folate levels in PBC patients compared to healthy controls. This reduction likely stems from multiple factors. First, chronic cholestasis in PBC can lead to maldigestion and anorexia, reducing dietary intake of folate-rich foods. Second, and perhaps more importantly, gut microbiota dysbiosis, a well-established feature of PBC, may compromise an important endogenous source of folate. Commensal bacteria such as *Bacteroides fragilis* and Bifidobacterium are capable of synthesizing folate (22). In patients with PBC, the reduced diversity and altered composition of the gut microbiota may impair this endogenous folate production (23). Lower expression of the folate biosynthesis pathway in cirrhotic versus non-cirrhotic PBC patients further strengthens the link between microbiota disruption, folate deficiency, and disease progression (23), suggesting that microbiota-mediated folate synthesis may further decline with disease progression.

The present study extends these findings by revealing a robust association between folate status and disease severity in PBC. Folate was significantly lower in late-stage PBC patients compared to those with early-stage disease and was negatively correlated with key biomarkers of liver injury (ALT, AST, GGT, IgG) and non-invasive fibrosis scores. These data suggest that folate deficiency is not merely a consequence of PBC but may actively contribute to its progression. Mechanistically, folate deficiency could exacerbate liver injury through several pathways. As a critical cofactor in one-carbon metabolism, folate deficiency impairs nucleotide synthesis and DNA repair, compromising hepatocyte regeneration. It also leads to homocysteine accumulation, which can promote oxidative stress, inflammatory mediator release, and hepatocyte apoptosis, manifesting as elevated transaminases (24). Furthermore, homocysteine can directly activate hepatic stellate cells (HSCs), driving collagen deposition and fibrogenesis (25), Our results align with previous studies showing that higher folate levels are associated with a lower risk of advanced liver fibrosis (26, 27). Finally, as a methyl donor, folate deficiency may influence the DNA methylation patterns (e.g., 5-mC and 5-hmC) critical for HSC activation and fibrogenesis (28).

To elucidate the molecular mechanisms through which folate deficiency contributes to the pathogenesis of PBC. We performed transcriptomic analysis on controls, normal-folate PBC patients, and low-folate PBC patients. The number of DEGs varied markedly across comparisons, with the most profound alterations observed in the low-folate PBC vs. control group (5,648 DEGs), followed by normal-folate PBC vs. low-folate PBC (2,343 DEGs) and normal-folate PBC vs. control (2,363 DEGs). This dose-dependent pattern of transcriptomic disturbance suggests that a low-folate state significantly disrupts hepatic gene expression homeostasis, and that these disruptions may be partially reversible with folate repletion. These findings provide preliminary molecular evidence for the protective role of folate in maintaining a stable hepatic transcriptome in PBC. Based on the transcriptomic screening, we selected six candidate genes—*ARID3B, MXD1, AHRR, P2RY14, DECR2, SPIB*—for protein-level validation by ELISA, yielding distinct expression patterns with potential functional implications for PBC pathogenesis.

ARID3B expression increased progressively from controls to normal-folate PBC to low-folate PBC, suggesting folate deficiency may upregulate ARID3B via methylation, promoting hepatocyte apoptosis and exacerbating PBC progression (29, 30). AHRR and MXD1 were significantly upregulated in PBC patients regardless of folate status (31, 32). AHRR may regulate Th17 immunity, while MXD1 likely modulates immune cell function or senescence, suggesting their involvement in PBC pathogenesis (33). P2RY14, a G protein-coupled receptor highly expressed in immune cells, regulates dendritic cell maturation and T lymphocyte proliferation, and has been implicated in autoimmune diseases such as ulcerative colitis (34). DECR2 is a peroxisomal enzyme involved in unsaturated fatty acid *β*-oxidation; its dysregulation may exacerbate cholestasis and hepatocyte injury (35).

In this study, ROC analysis showed that serum folate alone had moderate predictive value for advanced PBC (AUC = 0.652) with high specificity (90.9%), but combining folate with DBIL and LSM significantly improved diagnostic performance (AUC = 0.974). These findings suggest that low serum folate warrants heightened vigilance for disease progression, and that multi-marker panels are valuable for assessing PBC severity. Transcriptomic data also raise the possibility that folate supplementation may help restore gene expression homeostasis, though this requires prospective validation. Limitations include small sample size, cross-sectional design precluding causality, and protein validation in serum rather than liver tissue.

Several limitations of this study should be acknowledged. First, the sample size for RNA-seq analysis was relatively small (*n* = 7 per group), which may affect statistical power and generalizability. To mitigate this concern, we validated key differentially expressed genes using ELISA in an expanded independent cohort consisting of 38 PBC patients with normal folate, 38 PBC patients with low folate, and 38 healthy controls, and the protein-level findings were largely consistent with the transcriptomic results. Second, the retrospective cross-sectional design precludes causal inference; reverse causality, i.e., advanced PBC causing low folate levels due to malabsorption or malnutrition, cannot be ruled out. Third, a universal cutoff for defining low versus normal folate status is lacking. In this study, we adopted the lower limit of the normal reference range provided by the assay kit, but PBC-specific thresholds should be established in future studies. Fourth, other metabolites involved in folate-mediated one-carbon metabolism, such as vitamin B_12_ and homocysteine, were not measured, which limits a comprehensive mechanistic interpretation. Fifth, the patients’ response to UDCA was not evaluated, which could serve as a potential confounder when assessing disease progression. Sixth, the healthy control group lacked detailed information on dietary habits or folate supplementation, potentially introducing selection bias. Seventh, protein validation was performed in serum samples rather than in liver tissue, which may not fully capture intrahepatic molecular alterations. Despite these limitations, our multi-level approach provides novel insights into the role of folate deficiency in PBC progression and offers a foundation for future prospective and interventional studies.

In summary, this multi-level investigation demonstrates that low serum folate is significantly associated with PBC severity and may contribute to disease pathogenesis by disrupting hepatic gene expression homeostasis and modulating key genes involved in immune regulation, apoptosis, and lipid metabolism. The identified genes-*ARID3B, MXD1, AHRR, P2RY14, DECR-*represent promising candidates for understanding folate-mediated mechanisms in PBC and may serve as potential therapeutic targets. Our findings provide novel experimental evidence supporting the clinical value of monitoring folate levels in PBC patients and lay the groundwork for future studies investigating folate supplementation as an adjunctive therapeutic strategy.

## Data Availability

The original contributions presented in the study are included in the article/Supplementary material, further inquiries can be directed to the corresponding author.

## Supplementary material

SUPPLEMENTARY FIGURE 1 ROC curves for the predictive value of DBIL, FA, and LSM in advanced PBC.

## Glossary

PBC: Primary biliary cholangitis
ALT: Alanine aminotransferase
AST: Aspartate aminotransferase
GGT: *γ*-glutamyl transpeptidase
ALP: Alkaline phosphatase
TBIL: Total bilirubin
DBIL: Direct Bilirubin
IBIL: Indirect Bilirubin
ALB: Albumin
UA: Uric Acid
BUN: Blood Urea Nitrogen
SCr: Serum Creatinine
TC: Total Cholesterol
TG: Triglycerides
VLDL: Very low density lipoprotein
LDL-C: Low-Density Lipoprotein Cholesterol
HDL-C: High-Density Lipoprotein Cholesterol
PLT: Platelet
AMA-M2: Anti-mitochondrial Antibody M2 typing
gp210: Glycoprotein 210 antibody
UDCA: Ursodeoxycholic acid
IgA: Immunoglobulin A
IgG: Immunoglobulin G
IgM: Immunoglobulin M
CAP: Controlled Attenuation Parameter
LSM: Liver Stiffness Measurement
BMI: Body Mass Index
FA: Folic acid
AUC: Area Under the Curve
ROC: Receiver Operating Characteristic Curve
DEGs: Differentially expressed genes
DECR2: 2,4-dienoyl-CoA reductase 2
ARID3B: AT-rich interaction domain 3B
MXD1: MAX dimerization protein 1
AHRR: Aryl hydrocarbon receptor repressor
P2RY14: P2Y receptor family 14
SPIB: SPIB transcription factor

## References

[1] Tanaka A. Current understanding of primary biliary cholangitis. Clin Mol Hepatol. (2021) 27:1–21. doi: 10.3350/cmh.2020.0028, 33264835 PMC7820210

[2] Lleo A Leung PSC Hirschfield GM Gershwin EM. The pathogenesis of primary biliary cholangitis: a comprehensive review. Semin Liver Dis. (2020) 40:034–48. doi: 10.1055/s-0039-1697617 31537031

[3] Ronca V Davies SP Oo YH . The immunological landscape of primary biliary cholangitis: mechanisms and therapeutic prospects. Hepatology. (2025) 82:877–94. doi: 10.1097/HEP.0000000000001225, 39774114

[4] Trivella J John BV Levy C. Primary biliary cholangitis: epidemiology, prognosis, and treatment. Hepatol Commun. (2023) 7:e0179. doi: 10.1097/HC9.0000000000000179, 37267215 PMC10241503

[5] Lv T Chen S Li M Zhang D Kong Y Jia J. Regional variation and temporal trend of primary biliary cholangitis epidemiology: a systematic review and meta-analysis. J Gastroenterol Hepatol. (2021) 36:1423–34. doi: 10.1111/jgh.15329, 33141955

[6] Tanaka A Ma X Takahashi A Vierling JM. Primary biliary cholangitis. Lancet. (2024) 10:1168. doi: 10.1016/s0140-6736(24)01303-5, 39216494

[7] Levy C Manns M Hirschfield G. New treatment paradigms in primary biliary cholangitis. Clin Gastroenterol Hepatol. (2023) 21:2076–87. doi: 10.1016/j.cgh.2023.02.005, 36809835

[8] Wills L. Treatment of "pernicious anaemia of pregnancy" and "tropical anaemia" with special reference to yeast extract as a curative agent. Natl Med J India. (2013) 26:117–22. 24093997

[9] Ducker GS Rabinowitz JD. One-carbon metabolism in health and disease. Cell Metab. (2017) 25:27–42. doi: 10.1016/j.cmet.2016.08.009, 27641100 PMC5353360

[10] Lyon P Strippoli V Fang B Cimmino L. B vitamins and one-carbon metabolism: implications in human health and disease. Nutrients. (2020) 12:2867. doi: 10.3390/nu12092867, 32961717 PMC7551072

[11] Wang J Gao F Liu C Wang F. Association of folate levels with all-cause and cause-specific mortality in patients with arthritis. Clin Rheumatol. (2025) 44:953–68. doi: 10.1007/s10067-025-07337-8, 39853557

[12] Mangoni AA Zinellu A. Transsulfuration and folate pathways in rheumatoid arthritis: a systematic review and meta-analysis. Eur J Clin Investig. (2024) 54:e14158. doi: 10.1111/eci.14158, 38214126

[13] Da Mota J Carvalho LM Ribeiro AA da Mota JCNL Souza LL Borba EF . Methyl-donor supplementation in women with systemic lupus erythematosus with different nutritional status: the protocol for a randomised, double-blind, placebo-controlled trial. Lupus Sci Med. (2024) 11:e001279. doi: 10.1136/lupus-2024-001279, 39375179 PMC11459299

[14] Zhao M Tang J Gao F Wu X Liang Y Yin H . Hypomethylation of Il10 and Il13 promoters in Cd4+ T cells of patients with systemic lupus erythematosus. J Biomed Biotechnol. (2010) 2010:931018. doi: 10.1155/2010/931018, 20589076 PMC2879555

[15] Sun D Ma Y Bai Y Bai X Liu W du L . Folic acid combined with melatonin might prevent hepatic steatosis by alleviating endoplasmic reticulum stress to promote lipid droplet lipolysis in high-fat diet-fed mice. Nutrients. (2025) 17:3641. doi: 10.3390/nu17233641, 41373932 PMC12693466

[16] Yang M Wang D Wang X Mei J Gong Q. Role of folate in liver diseases. Nutrients. (2024) 16:1872. doi: 10.3390/nu16121872, 38931227 PMC11206401

[17] You H Duan W Li S Lv T Chen S Lu L . Guidelines on the diagnosis and management of primary biliary cholangitis (2021). J Clin Transl Hepatol. (2023) 11:736–46. doi: 10.14218/JCTH.2022.00347, 36969891 PMC10037524

[18] Al-Harthy N Kumagi T. Natural history and management of primary biliary cirrhosis. Hepatic Med Evid Res. (2012) 4:61–71. doi: 10.2147/HMER.S25998, 24367233 PMC3846599

[19] Trivedi PJ Hirschfield GM. Recent advances in clinical practice: epidemiology of autoimmune liver diseases. Gut. (2021) 70:1989–2003. doi: 10.1136/gutjnl-2020-322362, 34266966

[20] Medici V Halsted CH. Folate, alcohol, and liver disease. Mol Nutr Food Res. (2013) 57:596–606. doi: 10.1002/mnfr.201200077, 23136133 PMC3736728

[21] Li J Tian S Ci B Xi Y Deng X. Serum vitamins and homocysteine levels in autoimmune liver disease: a systematic review and meta-analysis. Immun Inflamm Dis. (2024) 12:e1258. doi: 10.1002/iid3.1258, 38652023 PMC11037259

[22] Hossain KS Amarasena S Mayengbam S. B vitamins and their roles in gut health. Microorganisms. (2022) 10. doi: 10.3390/microorganisms10061168, 35744686 PMC9227236

[23] Wang Q Tang X Qiao W Sun L Shi H Chen D . Machine learning-based characterization of the gut microbiome associated with the progression of primary biliary cholangitis to cirrhosis. Microbes Infect. (2024) 26:105368. doi: 10.1016/j.micinf.2024.105368, 38797428

[24] Fu L Wang Y Hu YQ. Association between homocysteine and nonalcoholic fatty liver disease: Mendelian randomisation study. Eur J Clin Investig. (2023) 53:e13895. doi: 10.1111/eci.13895, 36305497

[25] Ma C Zhang X Zhang W Duan J Yang H. Association between serum homocysteine levels and advanced hepatic fibrosis in alcohol-related liver disease: a cross-sectional study of Nhanes. Medicine (Baltimore). (2025) 104:e43395. doi: 10.1097/MD.0000000000043395, 40725931 PMC12303489

[26] Chen HK Luo J Li XJ Liao WZ Hu YQ Guo XG. Serum folate associated with nonalcoholic fatty liver disease and advanced hepatic fibrosis. Sci Rep. (2023) 13:12933. doi: 10.1038/s41598-023-39641-1, 37558738 PMC10412549

[27] Tripathi M Singh BK Zhou J Tikno K Widjaja A Sandireddy R . Vitamin B(12) and folate decrease inflammation and fibrosis in Nash by preventing syntaxin 17 homocysteinylation. J Hepatol. (2022) 77:1246–55. doi: 10.1016/j.jhep.2022.06.033, 35820507

[28] Page A Paoli P Moran Salvador E White S French J Mann J. Hepatic stellate cell transdifferentiation involves genome-wide remodeling of the Dna methylation landscape. J Hepatol. (2016) 64:661–73. doi: 10.1016/j.jhep.2015.11.024, 26632634 PMC4904781

[29] Wilsker D Probst L Wain HM Maltais L Tucker PW Moran E. Nomenclature of the arid family of Dna-binding proteins. Genomics. (2005) 86:242–51. doi: 10.1016/j.ygeno.2005.03.013, 15922553

[30] Tidwell JA Schmidt C Heaton P Wilson V Tucker PW. Characterization of a new arid family transcription factor (Brightlike/Arid3C) that co-activates bright/Arid3A-mediated immunoglobulin gene transcription. Mol Immunol. (2011) 49:260–72. doi: 10.1016/j.molimm.2011.08.025, 21955986 PMC3205283

[31] Hahn ME Sherr DH. The enigmatic Ahrr: beyond aryl hydrocarbon receptor repression. J Leukoc Biol. (2024) 116:915–8. doi: 10.1093/jleuko/qiae163, 39030724 PMC11531999

[32] Wang Q Wu Y Ouyang L Min X Zheng M Gao L . Single-cell analyses of intestinal epithelium reveal the dysregulation of gut immune microenvironment in systemic lupus erythematosus. J Transl Med. (2025) 23:118. doi: 10.1186/s12967-025-06147-5, 39871323 PMC11773722

[33] Grandori C Cowley SM James LP Eisenman RN. The Myc/max/mad network and the transcriptional control of cell behavior. Annu Rev Cell Dev Biol. (2000) 16:653–99. doi: 10.1146/annurev.cellbio.16.1.653, 11031250

[34] Cekic C Linden J. Purinergic regulation of the immune system. Nat Rev Immunol. (2016) 16:177–92. doi: 10.1038/nri.2016.4, 26922909

[35] Alphey MS Yu W Byres E Li D Hunter WN. Structure and reactivity of human mitochondrial 2,4-dienoyl-CoA reductase: enzyme-ligand interactions in a distinctive short-chain reductase active site. J Biol Chem. (2005) 280:3068–77. doi: 10.1074/jbc.M411069200, 15531764

