# Supplementary figures and images for: Folate deficiency correlates with severity of primary biliary cholangitis via modulating key regulatory genes
Source: Front Nutr. 2026 Jun 3;13:1838352. doi: 10.3389/fnut.2026.1838352 (PMC13271935; doi:10.3389/fnut.2026.1838352)

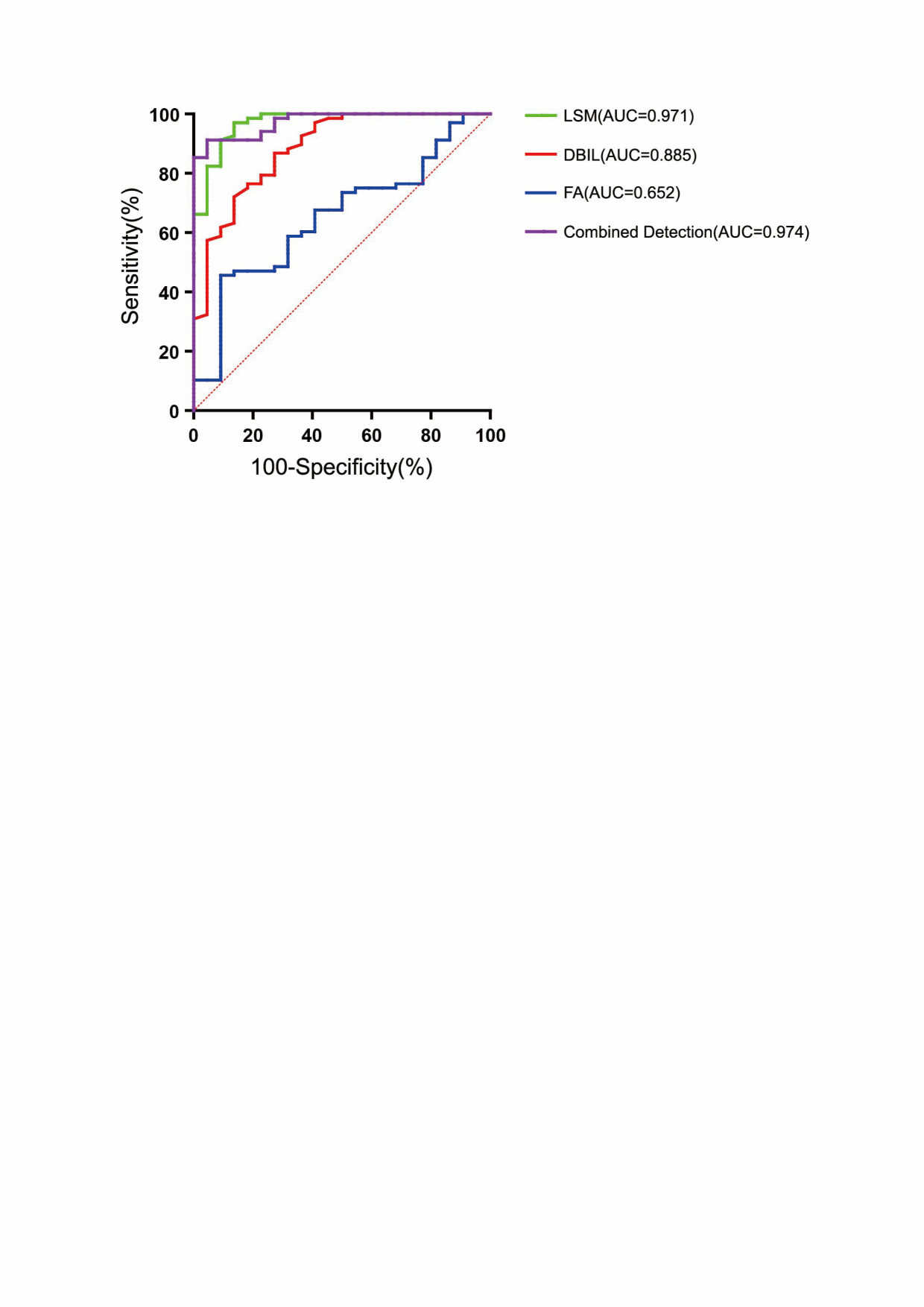

Supplement: SUPPLEMENTARY FIGURE 1 — ROC curves for the predictive value of DBIL, FA, and LSM in advanced PBC. [file Image_1.TIFF]
